# Plant immunity inducers: from discovery to agricultural application

**DOI:** 10.1007/s44154-021-00028-9

**Published:** 2022-01-10

**Authors:** Bo Yang, Sen Yang, Wenyue Zheng, Yuanchao Wang

**Affiliations:** 1 Department of Plant Pathology, Nanjing Agricultural University, Nanjing, 210095 China; 2 The Key Laboratory of Plant Immunity, Nanjing Agricultural University, Nanjing, 210095 China

**Keywords:** Plant immunity inducer, Plant immunity, Biopesticide, Agricultural applications

## Abstract

While conventional chemical fungicides directly eliminate pathogens, plant immunity inducers activate or prime plant immunity. In recent years, considerable progress has been made in understanding the mechanisms of immune regulation in plants. The development and application of plant immunity inducers based on the principles of plant immunity represent a new field in plant protection research. In this review, we describe the mechanisms of plant immunity inducers in terms of plant immune system activation, summarize the various classes of reported plant immunity inducers (proteins, oligosaccharides, chemicals, and lipids), and review methods for the identification or synthesis of plant immunity inducers. The current situation, new strategies, and future prospects in the development and application of plant immunity inducers are also discussed.

## Introduction

Plant diseases are responsible for substantial crop losses each year; they are threats to global agricultural sustainability and food security (Oerke and Dehne 2004). People have generally expected thorough and rapid elimination of disease-causing plant pathogens via chemical pesticide application. However, the widespread and unregulated use of chemical pesticides increases the cost of agricultural production; it also causes problems such as environmental pollution, excessive pesticide residues in agricultural products, and pathogen resistance. These problems seriously restrict the sustainable development of agriculture and directly endanger human health. To address the current food safety and environmental pollution crisis, there is an urgent need to identify new methods and technologies for plant protection that are economical, efficient, safe, and environmentally friendly. Plants normally remain healthy despite exposure to different microbes because they have evolved a multilayered immune system that can recognize all classes of pathogens. With advancements in science and technology, the mechanisms of plant immunity have been gradually revealed; the principles of plant immunity have been widely used in the prevention and control of crop diseases and insect pests (Dangl et al. 2013; Zipfel 2014). Biological factors that can activate plant immunity are collectively known as plant immunity inducers; these are present in many microorganisms, plants, and animals. In addition to breeding disease-resistant crops via disease resistance receptors, the improvement of crop resistance to pathogens using plant immunity inducers is an environmentally sound method for managing disease. In this review, we focus on plant immunity inducers, including their identification methods, applications, and related mechanisms. We also discuss the current status and potential future developments of plant immunity inducers.

### Recognition of plant immunity inducers

In the initial stage of the interaction between pathogens and plants, pathogens must break through the physical barriers on the plant surface; they must also overcome a plant’s active defense system to achieve successful colonization (Jones and Dangl 2006). During the early stage of infection, pathogens secrete some virulence factors to attack plants; plants gradually evolved membrane surface pattern recognition receptors to recognize the biochemically conserved pathogen-associated molecular patterns (PAMPs) of pathogens, which enable the activation of PAMP-triggered immunity (PTI). PTI is a ubiquitous but weak resistance that can temporarily prevent infection by most pathogens (Jones and Dangl 2006). In turn, pathogens have evolved diverse virulence factors (i.e., effectors) that can be delivered into host cells to interfere with PTI and dampen basal defenses (Jones and Dangl 2006; Wang et al. 2019b). In response, plants have developed an additional layer of defense that enables them to intercept pathogen effectors, leading to effector-triggered immunity. This layer of plant defense is driven by a family of polymorphic intracellular nucleotide-binding/leucine-rich repeat receptors. However, adaptive plant pathogens have gained the ability to overcome effector-triggered immunity by effector variation or the secretion of new effectors (Jones and Dangl 2006). Compared to PTI, effector-triggered immunity leads to stronger resistance, usually accompanied by the hypersensitive response, a form of programmed cell death at the site of infection. However, the large-scale use of a single disease-resistant variety in the field will increase selection pressure among pathogens, thus accelerating their evolution; such changes are not conducive to the long-term prevention and control of plant diseases. The induced resistance of plants makes full use of the potential ability for plants themselves to prevent disease; it induces a basic immune response by regulating plant defense and metabolic systems to delay or mitigate disease occurrence and development.

Plant pattern recognition receptors that recognize PAMPs include receptor-like kinases (RLKs) and receptor-like proteins (RLPs) (Shiu and Bleecker 2003; Shiu et al. 2004). RLKs are located on the plasma membrane and contain an extracellular ligand binding domain, a transmembrane domain, and intracellular protein kinase domain. By contrast, RLPs only contain extracellular and transmembrane domains; they lack intracellular kinase domains. Thus, RLPs must bind to other intracellular co-receptor kinases to transmit downstream signals (Boutrot and Zipfel 2017). Although many plant immunity inducers and their corresponding recognition receptors have been identified, the induction of plant immunity by most elicitors remains poorly understood. The best-characterized PAMPs are bacterial flagellin (recognized by plant flagellin-sensing 2 [FLS2]) and bacterial elongation factor Tu (EF-Tu; recognized by the LRR-RLK EF-Tu receptor) (Gomez-Gomez and Boller 2000; Zipfel et al. 2006). Flagellin is a structural protein of a bacterial flagellum, which extends from the cell surface and allows bacteria to be motile. Flg22, a conserved 22-amino acid epitope of the N terminus of flagellin, is sufficient to activate an immune response. However, flg22 is buried in the flagellin polymer structure, such that it cannot bind to FLS2 (Fliegmann and Felix 2016). A recent study revealed that plants use glycosidase to facilitate the release of flg22 from invading bacteria (Buscaill et al. 2019). FLS2 homologous genes have been found in all higher plants with known genomic information. Proteins homologous to FLS2 in tobacco, rice, and tomato recognize bacterial flagellin. These results indicate that plant FLS2 proteins function as evolutionarily conserved and ancient receptors to recognize flagellin (Felix et al. 1999; Gomez-Gomez and Boller 2000).

The recognition of plant immunity inducers by plant receptors is only the beginning of the immune response. Pattern recognition receptors must work in conjunction with other plasma membrane proteins to transmit immune signals downstream through the corresponding signal transduction pathway, enabling plants to perform additional immune response functions (Liang and Zhou 2018). BRI1-associated receptor kinase 1 (BAK1) is a co-receptor protein that participates in various signaling pathways; it is involved in PTI signaling mediated by multiple PAMPs receptor complexes (Chinchilla et al. 2009). After the activation of plant pattern recognition receptors, the signal is usually transmitted downstream by protein phosphorylation; it causes disease-related immune responses such as downstream Ca^2+^ signal inflow, reactive oxygen accumulation, callose accumulation, stomatal closure, and salicylic acid production (Liang and Zhou 2018; Tang et al. 2017; Zhou and Zhang 2020).

### Major classes of plant immunity inducers

Plant immunity inducers can be derived from animals, plants, microbes or their metabolites, active molecules produced during interactions between plants and microbes, or natural/synthetic compounds. According to their chemical properties, plant immunity inducers can be classified into proteins, oligosaccharides, glycopeptides, lipids, lipopeptides, small molecule metabolites, and chemical compounds (Boutrot and Zipfel 2017; Schwessinger and Ronald 2012). In this review, we have listed a wide array of plant immunity inducers that have been shown to induce PTI-like responses in plants (Table 1). The identification of these components has accumulated considerable resources for the development of plant immune-induced pesticides.

**Table 1:** The major classes of plant immune inducers

Attributes	Names	Origin	Refs.
Proteins	Harpin	*Pseudomonas syringae*	(Dong et al. 1999)
	Siderophore	*Pseudomonas fluorescens*	(Leeman et al. 1996)
	Flagellin	*Pseudomonas syringae*	(Felix et al. 1999)
	Nep1-like protein	*Bacillus halodurans*	(Oome et al. 2014)
	AMEP412	*Bacillus subtilis*	(Shen et al. 2019)
	SodM	*Escherichia coli*	(Watt et al. 2006)
	EF-Tu	*Escherichia coli*	(Kunze et al. 2004)
	PGN	*Staphylococcus aureus*	(Gust et al. 2007)
	CSP	*Staphylococcus aureus*	(Felix and Boller 2003)
	PeBL1	*Brevibacillus laterosporus*	(Wang et al. 2015)
	EG1(GH45)	*Rhizoctonia solani*	(Ma et al. 2015a)
	EIF	*Trichoderma viride*	(Fuchs et al. 1989)
	Nip1	*Rhynchosporium commune*	(Rohe et al. 1995)
	MgSM1	*Magnaporthe grisea*	(Yang et al. 2009)
	PemG1	*Magnaporthe oryzae*	(Peng et al. 2011)
	Ave1	*Verticillium dahliae*	(de Jonge et al. 2012)
	PevD1	*Verticillium dahliae*	(Wang et al. 2012a)
	PebC1	*Botrytis cinerea*	(Zhang et al. 2014c)
	BcGs1(GH15)	*Botrytis cinerea*	(Zhang et al. 2015)
	BcIEB1	*Botrytis cinerea*	(Frias et al. 2016)
	BcPGs (GH28)	*Botrytis cinerea*	(ten Have et al. 1998)
	T4BcPG1	*Botrytis cinerea*	(Poinssot et al. 2003; Zhang et al. 2014b)
	Hrip	*Alternaria tenuissima*	(Kulye et al. 2012)
	AsES	*Acremonium strictum*	(Chalfoun et al. 2013)
	SsCut	*Sclerotinia sclerotiorum*	(Zhang et al. 2014a)
	Cyclodipeptides	*Eupenicillium brefeldianum*	(Chen et al. 2015)
	CS20EP	*Fusarium oxysporum*	(Shcherbakova et al. 2016)
	RALF	*Fusarium oxysporum, Arabidopsis*	(Pearce et al. 2001; Thynne et al. 2017)
	Six4	*Fusarium oxysporum*	(Houterman et al. 2008)
	Six1	*Fusarium oxysporum*	(Rep et al. 2004)
	SnTox1	*Stagonospora nodorum*	(Liu et al. 2012b)
	PB90	*Phytophthora boehmeriae*	(Wang et al. 2003)
	Elicitin	*Phytophthora capsici*	(Ricci et al. 1989)
	OPEL (GH16)	*Phytophthora parasitica*	(Chang et al. 2015)
	GP42	*Phytophthora sojae*	(Nurnberger et al. 1994)
	AEP1	*Phytophthora sojae*	(Xu et al. 2021)
	PC2	*Phytophthora infestans*	(Wang et al. 2021)
	SCR96	*Phytophthora cactorum*	(Chen et al. 2016)
	CBEL	*Phytophthora parasitica*	(Gaulin et al. 2006)
	PcF	*Phytophthora cactorum*	(Orsomando et al. 2001)
	XEG1	*Phytophthora sojae*	(Ma et al. 2015b)
	GH17	*Cladosporium fulvum*	(Ökmen et al. 2019)
	NLP	*Phytophthora parasitica*	(Böhm et al. 2014)
	TaMCA4	*Puccinia striiformis* f. sp. *tritici*	(Wang et al., 2012b)
	Coat protein	Tobacco mosaic virus	(Allan et al. 2001)
	Gr-VAP1	*Heterodera rostochiensis*	(Lozano-Torres et al. 2012)
	VmE02	*Valsa mali*	(Nie et al. 2019)
	VdCP1	*Verticillium dahliae*	(Zhang et al. 2017)
	VdPEL1	*Verticillium dahliae*	(Yang et al. 2018)
	AGLIP1	*Rhizoctonia solani*	(Li et al. 2019)
	BAR11	*Saccharothrix yanglingensis*	(Zhang et al. 2018b)
	CfPDIP1	*Colletotrichum falcatum*	(Ashwin et al. 2018)
	Fg12	*Fusarium graminearum*	(Yang et al. 2021)
	Systemin	Tomato	(Pearce et al. 1991)
	ATP synthase	Maize	(Schmelz et al. 2006)
Peptide	Phytosulfokine	Tomato	(Zhang et al. 2018a)
	AtPep1	Arabidopsis	(Huffaker et al. 2006)
	PIPs	Arabidopsis	(Hou et al. 2014)
	GmPeps	Soybean	(Lee et al. 2018; Pearce et al. 2010; Yamaguchi et al. 2011)
Carbohydrates	Exopolysaccharides	*Xanthomonas campestris* pv. *vesicatoria*	(Romeiro and Kimura 1997)
	Chitin	*Agaricus bisporus*	(Kaku et al. 2006)
	Oligochitosan	*Fusarium solani*	(Cabrera et al. 2006)
	Heptaglucoside	*Phytophthora sojae*	(Sharp et al. 1984)
	Xyloglucan	*Rubus fruticosus*	(Joseleau et al. 1992)
	D-allose	*Oryza sativa*	(Kano et al. 2010)
	Galactinol	Tobacco	(Kim et al. 2008)
	Trehalose	Arabidopsis	(Reignault et al. 2001)
	Laminarin	Arabidopsis	(Ménard et al. 2004)
	Lichenan	Tobacco	(Stübler and Buchenauer 1996)
	Mannan oligosaccharides	Carob	(Zang et al. 2019)
Lipids/Lipopeptides	Lipopolysaccharides	*Staphylococcus aureus*	(Dow et al. 2000)
	N-acyl-homoserine lactones	*Serratia liquefaciens*	(Schuhegger et al. 2006)
	Fatty acid amides	*Spodoptera exigua*	(Alborn et al. 1997)
	Surfactin	*Bacillus amyloliquefaciens*	(Rahman et al. 2015)
	Ergosterol	*Cladosporium fulvum*	(Granado et al. 1995)
	Fengycin	*Bacillus amyloliquefaciens*	(Farzand et al. 2019)
	Iturin	*Bacillus amyloliquefaciens*	(Han et al. 2015)
	Lokisin	*Pseudomonas* sp. *COR10*	(Omoboye et al. 2019)
	Eicosapentaenoic acid	*Phytophthora infestans*	(Bostock et al. 1981)
	Arachidonic acid	*Phytophthora infestans*	(Savchenko et al. 2010)
	Cerebroside	*Magnaporthe oryzae*	(Koga et al. 1998)
	Rhamnolipids	*Pseudomonas aeruginosa*	(Crouzet et al. 2020; Sanchez et al. 2012)
Nucleotides	Extracellular ATP	Arabidopsis	(Clark et al. 2011; Tanaka et al. 2014)
	Extracellular pyridine nucleotides		(Wang et al. 2019a)
Chemical	DL-β-aminobutyric acid		(Hong et al. 1999)
	Benzothiadiazole		(Oostendorp et al. 2001)
	2,6-dichloro isonicotinic acid		(Qian et al. 2006)
	Isotianil		(Kumar et al. 2018)
	Methiadinil		(Wang et al. 2017)
	Probenazole		(Yoshioka et al. 2001)
	Dufulin		(Li and Song 2017)
	4-fluorophenoxyacetic acid		(Wang et al. 2020)
	3-pentanol	*Bacillus amyloliquefaciens*	(Choi et al. 2014a)
	Pipecolic acid	Plants	(Návarová et al. 2012)
	2,4-diacetyl phloroglucinol	*Pseudomonas fluorescens*	(Chae et al. 2020)
	2,3-butanediol	*Pseudomonas chlororaphis*	(Kong et al. 2018)
	Hexadecane	*Paenibacillus polymyxa*	
	Tridecane	*Paenibacillus polymyxa*	(Lee et al. 2012)

#### Plant immunity-inducing proteins

In recent years, many plant immunity-inducing proteins, such as flagellin (Felix et al. 1999), harpin (Dong et al. 1999), nepl-like protein (Oome et al. 2014), xylanase (Fuchs et al. 1989), elicitin (Ricci et al. 1989), cellulose (Ma et al. 2015a), RNAse (Yang et al. 2021), and aldose 1-epimerase (Xu et al. 2021) have been identified in bacteria, fungi, oomycetes, viruses, and plants (Table 1). Here, we highlight several recently discovered plant immunity-inducing proteins. PsXEG1 is an apoplastic xyloglucan-specific endoglucanase that is secreted by soybean root rot pathogen *Phytophthora sojae*; it belongs to the glycoside hydrolase GH12 family (Ma et al. 2015b). It can promote *P*. *sojae* infection via plant cell wall degradation. Furthermore, PsXEG1-like GH12 proteins are widely distributed in *Phytophthora*, many pathogenic fungi, and bacteria. PsXEG1 can induce multiple plant immune responses, including oxidative burst, callose accumulation, robust expression of disease-resistant genes, and the hypersensitive response in plants (Ma et al. 2015b; Ma et al. 2017). Notably, PsXEG1 induces immune responses in soybean plant, which is the host plant of *P*. *sojae*; it also elicits cell death in non-host plants such as tobacco, tomato, and pepper, indicating that plants recognize PsXEG1 using a conserved mechanism. The recognition of XEG1 in plants depends on the RLP kinase protein BAK1, which is a co-receptor that transmits immune signals via cooperation with other receptor proteins (Ma et al. 2015b). A high-throughput LRR receptor-like gene silencing library was established in *Nicotiana benthamiana*, which can efficiently silence 386 LRR receptor-like genes. NbRXEG1, a receptor-like protein that is responsible for the recognition of PsXEG1, was successfully identified via screening of this library (Wang et al. 2018). NbRXEG1 can specifically bind to PsXEG1, thereby activating the corresponding immune pathway (Wang et al. 2018). Fg12, a ribonuclease secreted by *Fusarium graminearum*, induces cell death in *N*. *benthamiana* in a light-dependent manner; *F*. *graminearum* pathogenesis depends on the ribonuclease activity of Fg12 (Yang et al. 2021). *P*. *sojae*-secreted aldose 1-epimerase triggers cell death in plants such as tobacco, tomato, potato, eggplant, pepper, and Arabidopsis. The enzymatic activity of aldose 1-epimerase is dispensable for its cell death-inducing activity, while aldose 1-epimerase-mediated immune signaling in *N*. *benthamiana* requires BAK1 (Xu et al. 2021). Many other microbe-derived plant immunity-inducing proteins (e.g., glycosyl hydrolase GH17 protein, lipase, and some proteins without known functional domains) also exhibit robust plant immunity-inducing activity.

In addition to microbe-derived plant immunity inducers, some plant-derived elicitors activate plant defense responses. For instance, a universally conserved plant-derived elicitor is Pep1, a conserved epitope derived from the C terminus of PROPEP1 (Huffaker et al. 2006). Pep1 is recognized on the cell surface by the PEPRs, leading to the induction of immune responses (Krol et al. 2010; Yamaguchi et al. 2010). One study has provided excellent proof that metacaspase MC4 is responsible for PROPEP1 cleavage and the release of Pep1 (Hander et al. 2019). Other plant-derived elicitors, such as GmPep914 and GmSubPep (two peptides identified in soybean) can effectively induce plant resistance to different pathogens (Pearce et al. 2010; Yamaguchi et al. 2011).

#### Plant immunity-inducing carbohydrates

There are abundant plant immunity-inducing carbohydrates in nature. Most are derived from the shell or cell wall of animals, plants, or microbes; they include exopolysaccharides, chitin, xyloglucan, and oligochitosan (Table 1) (Cabrera et al. 2006; Kaku et al. 2006; Romeiro and Kimura 1997; Sharp et al. 1984). Mannan oligosaccharide, which is hydrolyzed from locust bean gum, significantly enhances the generation of intracellular Ca^2+^ and reactive oxygen species, as well as the accumulation of four phytoalexins (Zang et al. 2019). Treatment with mannan oligosaccharide conferred resistance in rice and tobacco against *Xanthomonas oryzae* and *Phytophthora nicotianae*. Laminarin is a water-soluble β-1,3-glucan that can induce defense responses in plants such as tobacco, Arabidopsis, and rice (Klarzynski et al. 2000). It can induce immune pathways that rely on the salicylic acid signaling pathway, including the accumulation of phytoalexins and expression of various disease-related proteins (Klarzynski et al. 2000). Trehalose is a non-reducing disaccharide widely distributed in many species. The treatment of wheat with trehalose significantly increases wheat resistance to powdery mildew by increasing the enzymatic activity of phenylalanine ammonia lyase and peroxidase, which are related to the wheat defense response (Reignault et al. 2001). Chitin, a major component in the cell wall of filamentous fungi, induces defense responses in various plants. OsCEBiP and AtLYK5, two receptors identified in rice and *Arabidopsis thaliana*, respectively, have been described as receptors that directly bind to chitin (Hayafune et al., 2014; Cao et al., 2014). CERK1, a receptor-like kinase containing a lysozyme structure, also has an important role in chitin recognition (Cao et al., 2014; Petutschnig et al. 2010). In addition, the receptor-like proteins AtLYM2, OsLYP4, and OsLYP6 are required for responses to peptidoglycan and chitin (Faulkner et al. 2013; Liu et al. 2012a).

#### Plant immunity-inducing lipids

Currently, the known plant immunity-inducing lipids mainly include lipopolysaccharide, ergosterol, eicosapentaenoic acid, and arachidonic acid (Bostock et al. 1981; Dow et al. 2000; Granado et al. 1995; Savchenko et al. 2010). Lipopolysaccharide is a component of the cell wall of Gram-negative bacteria; it is essential for bacterial infection (Dow et al. 2000). It can also be recognized by various plants and induce plant immune responses. Medium-chain 3-hydroxy fatty acids, widely present in Gram-negative bacteria, are reportedly recognized by a lectin S-domain receptor kinase LORE (Kutschera et al., 2019). Ergosterol, a component extracted from fungus, induces the extracellular alkalization of tomato cells and activates immune responses in tomatoes, requiring only a low concentration (Granado et al. 1995). Similarly, arachidonic acid and eicosapentaenoic acid from *Phytophthora infestans* can activate immune responses in potato (Bostock et al. 1981; Savchenko et al. 2010). Fatty acid amides, isolated from the oral secretions of *Spodoptera exigua* caterpillar larvae, induce corn seedlings to emit volatile compounds that attract natural enemies of the caterpillars (Alborn et al. 1997).

#### Plant immunity-inducing chemical compounds

Chemical compounds such as benzothiadiazole, 2,6-dichloro isonicotinic acid, probenazole, and dufulin are elicitors that activate plant immunity. For example, benzothiadiazole induces plant resistance to numerous diseases, consistent with its resemblance to salicylic acid (Thakur and Sohal 2013). DL-β-aminobutyric acid can induce local and systemic resistance to *Colletotrichum coccodes* in pepper plants (Hong et al. 1999). Isotianil, which was identified by Bayer AG and Sumitomo Chemical Co., Ltd., induces the expression of plant pathogenesis-related genes; it can efficiently activate rice resistance to rice blast disease (Kumar et al. 2018). Dufulin enhances defense enzyme activity, increases the chlorophyll content in tobacco leaves, and prevents tobacco viral diseases (Li and Song 2017). Recently, 4-fluorophenoxyacetic acid has been shown to suppress white-backed planthopper *Sogatella furcifera* populations and increase crop yields in the field (Wang et al. 2020). 4-Fluorophenoxyacetic acid modulates the production of peroxidases, H_2_O_2_, and flavonoids; it directly triggers the formation of flavonoid polymers, thereby increasing the resistance of cereals to piercing-sucking insect pests (Wang et al. 2020).

#### Other types of plant immunity inducer

In addition to the inducers described above, substances such as extracellular ATP and cerebroside can activate plant immunity. For example, at a particular concentration, extracellular ATP from plants can induce stomata closure. The receptor-like kinase DORN1 is the receptor of extracellular ATP in plants. The recognition of extracellular ATP by DORN1 induces immune responses such as calcium burst and mitogen-activated protein kinase activation in plants (Choi et al. 2014b).

### Strategies for identification or chemosynthesis of plant immunity inducers

#### Biochemical approaches to identify plant immunity inducers

The most commonly used and most effective method for identification of plant immunity inducers is isolation and purification from the culture extracts of microorganisms via biochemical approaches. Single components are separated from cultures by ion exchange chromatography and high-performance liquid chromatography; the compositions of those single components are then identified by mass spectrometry. Many plant immune elicitors have been successfully identified by this method, such as flagellin from *Pseudomonas syringae* (Felix et al. 1999) and glycosyl hydrolase PsXEG1 from *P*. *sojae* (Ma et al. 2015b). In addition, nuclear magnetic resonance spectroscopy and fast atom bombardment analysis have been employed to identify cerebroside from *Magnaporthe oryzae* (Koga et al. 1998). Finally, the use of known receptor proteins as molecular bait is a promising approach to identify plant immunity inducers that can specifically bind to those receptor proteins.

#### Sequence analysis to identify plant immunity inducers

Comparison of the sequence similarity of existing plant immunity inducers among species is an effective method to identify new plant immunity inducers. For example, necrosis- and ethylene-inducing peptide (NLP) protein was initially identified in the pathogenic fungus *Fusarium oxysporum* (Bailey 1995). Through similarity comparison, multiple NLP (Nep1-like) proteins were identified in bacteria, fungi, and oomycetes (Oome et al. 2014; Qutob et al. 2002). Recent studies have shown that the 24 amino acids (nlp24) conserved in the NLP protein are sufficient to induce immune responses in plants; the NLP receptor RLP23 has also been identified in *A. thaliana* (Albert et al. 2015). Many other variants of plant immunity inducers have been identified through sequence homology analysis, such as oomycete elicitin and bacterial flagellin (Derevnina et al. 2016).

#### Chemosynthesis of plant immunity inducers

Advances in combinatorial chemistry and the development of high-throughput screening systems have enabled scientists to conduct comprehensive assays for the identification of synthetic plant immunity inducers. Simple chemical derivation of known plant immunity inducers is an efficient method to design more potent immune elicitors (Zhou and Wang 2018). The combination of a known synthetic plant immunity inducer with another functional compound is also effective for the generation of bifunctional product. For example, the combination of 3,4-dichloroisothiazoles with fungicidal strobilurins produced a new synthetic compound with good fungicide activities (Chen et al. 2017). Moreover, the computer-aided design compound design strategy has been utilized for pesticide discovery and property analysis. Thus, applications of computer-aided design can generate new plant immunity inducers.

### Applications of plant immunity inducers

In recent years, the development and generation of plant immunity inducers have been conducted based on known plant immunity elicitors. A notable example is the harpin protein isolated from the plant pathogen *Erwinia amylovora* by Cornell University and Eden Biotechnology (Wei et al. 1992). The harpin protein has a unique immune-activating function; it has been developed into a protein biological pesticide: Messenger. After years of application, Messenger has become a world-renowned immunity inducer with protective activity against numerous plant diseases. This achievement has won the “Presidential Green Chemistry Challenge Award” issued by the Environmental Protection Agency. It has been widely used in the growth of fruit, tobacco, and vegetables. In addition, researchers from the Institute of Plant Protection of the Chinese Academy of Agricultural Sciences have isolated and purified plant immunity-inducing proteins such as PeaT1 and Hrip1 from *Alternaria tenuissima* and other pathogenic fungi (Kulye et al. 2012). ATaiLing, which mainly consists of plant immunity-inducing proteins and oligosaccharides, was approved for pesticide application in 2014. Field trials showed that its prevention and control effects against crop virus disease were greater than 70%, and the crop yield was increased by more than 10%; it received considerable praise from farmers after release to the broader market (Qiu 2016). Researchers from China Agricultural University identified the immune-activating protein VdAL in *Verticillium dahlia*; they showed that the treatment of cucumber seedlings with VdAL led to significant resistance enhancement and improved the storage quality of cucumber commercial seedlings (Sun et al. 2016). ZhiNengCong, an extract of the fungal endophyte *Paecilomyces variotii*, has been widely used in China (Lu et al. 2019). ZhiNengCong functions as an elicitor to both induce plant resistance and promote crop growth (Lu et al. 2019).

Many research groups have made considerable progress in the development of plant immunity-inducing oligosaccharides. For example, the Dalian Institute of Chemical Physics, Chinese Academy of Sciences has made important breakthroughs in identifying applications of oligosaccharides. In particular, chitosan oligosaccharides have been used as raw materials to develop plant immunity-inducing oligosaccharides; these oligosaccharides received multiple approvals for pesticide application and provided considerable improvements in crop yield and quality (Qiu 2016). In addition, Korea Ukseung Chemical Co., Ltd. developed a chitosan product, with an active ingredient of chitosan oligosaccharide, which is derived from the shells of shrimp, crab, and other organisms. The treatment of crop seeds with this agent significantly prevented disease and increased yields. Chitosan-oligosaccharide biologics have been certified as environmentally friendly crop production active agents by the Ministry of Agriculture and Forestry of Korea. Moreover, oligochitosan can stimulate defense responses in rice leaves, increasing the production of phenolic secondary metabolites to enhance resistance against rice blast disease (Agrawal et al. 2002). The treatment of wheat with oligochitosan promoted resistance to *Bipolaris sorokiniana* (Burkhanova et al. 2007). In addition to applications in crop plants, oligochitosan has been shown to induce tobacco resistance to *Phytophthora parasitica* and potato resistance to *Phytophthora infestans* (Falcón et al. 2008; Ozeretskovskaya et al. 2006). Researchers have also developed various plant immunity-inducing chemical compounds, such as dufulin, which was generated by a research team from Guizhou University. Isotianil, an isothiazole-based synthetic plant immunity inducer, has been widely used in Japan to control rice disease.

### Promising new strategies for the utilization of plant immunity inducers

After the identification of plant immunity inducers from plants or microorganisms, large-scale utilization remains an important challenge. Ectopic expression of the genes that encode elicitors in plants is also an effective strategy. For instance, the overexpression of harpin-encoding genes confers enhanced resistance to pathogens in several transgenic plants (e.g., soybean, rice, tobacco, and sugar beet) (Du et al. 2018; Li et al. 2012; Pavli et al. 2011). In addition to using transgenic plants, engineered bacteria are often used to produce plant immunity inducers; for example, harpin protein was industrially produced in a genetically engineered strain of *Escherichia coli* (Bauer et al. 1997; Wei et al. 1992). However, the target proteins must be released from bacterial cells by sonication or high pressure; the protein purification step is expensive. In addition, during the production and purification of proteins in *E. coli*, there are problems such as inefficient gene expression, protein inclusion body formation, and bacterial toxin production. Thus, microbes that secrete proteins directly into the fermentation broth offer major advantages for downstream production processing. *Bacillus* species are excellent microorganisms for use in secreted protein production. *Bacillus subtilis* is one of the best-characterized Gram-positive bacteria; it has been widely used because of its high product yields, its complete lack of toxic by-products, and its excellent fermentation properties (van Dijl and Hecker 2013). For example, *B. subtilis*-mediated expression of the HpaG_Xooc_ protein, which is a component of the harpin protein from *X. oryzae* pv. *oryzicola*, elicits the hypersensitive response and enhances growth in tobacco (Wu et al. 2009). Moreover, *B. subtilis* is classified as “generally recognized as safe” by the US Food and Drug Administration; several *B. subtilis* strains promote plant growth and increase plant resistance. Recently, a new strategy was proposed to manage plant diseases by combining two effective methods: use of the plant Pep elicitor to enhance crop resistance to plant-parasitic nematodes and use of *B. subtilis* for efficient delivery of these elicitors (Hiltl and Siddique 2020; Zhang and Gleason 2020). This strategy will provide new avenues for the combined application of plant immunity inducers and beneficial microorganisms to protect plant health.

### Prospects for the development of plant immunity inducers

The development of plant immunity inducers is a new field of plant immunity engineering technology. This popular new field enables scientists to scientifically control pests and diseases based on plant immunity theory, supporting biopesticide generation; it is expected to become a new strategic industry with extensive development prospects (Qiu et al. 2017). Compared to chemical pesticides, plant immunity inducers have many advantages, such as their exceptionally low dosage, lack of damaging effects for humans and animals, lack of adverse effects on the environment, ability to induce substantial disease resistance, induction of long-term and broad-spectrum plant resistance, low risk for microbial resistance, and ability to reduce the use of chemical pesticides—this reduction can aid in environmental protection efforts. In recent years, the Chinese government has increasingly focused on environmental protection; it has prioritized the construction of ecological civilization. The government vigorously promotes the reduction of pesticides and fertilizers; it also advocates for increased efficiency and the active exploration of efficient, safe, and environmentally friendly modern agricultural development. The identification, development, and application of plant immunity inducers meet the strategic needs of national development; it has important academic value and can serve agricultural production, facilitating important contributions to ensure widespread food safety and sustainable agriculture development.

Regarding the research and application of future plant immunity inducers, there are several important considerations. First, a system must be constructed for the screening, functional analysis, and evaluation of the activities of plant immunity inducers, which must be systematically identified from different sources. Second, there is a need to investigate plant recognition receptors, key recognition sites, and signaling pathways of plant immunity inducers to identify the corresponding mechanisms. The identification of principal compounds that elicit resistance is an important aspect that must be considered. Third, new technologies and processes are needed for the large-scale production of plant immunity inducers, thereby reducing the cost of their industrialization. Fourth, the rational combination of plant immunity inducers and chemical pesticides, beneficial microorganisms, or fertilizer is needed for synergistic enhancement of plant resistance and production. Finally, synthetic compounds should be designed based on the structures of plant receptors and their key sites for recognition; these efforts will promote the generation of plant immunity inducers.

## Data Availability

Not applicable.
